# Diversity, mechanisms and beneficial features of phosphate-solubilizing *Streptomyces* in sustainable agriculture: A review

**DOI:** 10.3389/fpls.2022.1035358

**Published:** 2022-12-06

**Authors:** Fatima Ezzahra Chouyia, Valeria Ventorino, Olimpia Pepe

**Affiliations:** ^1^ Department of Biology, Faculty of Sciences and Techniques, Hassan II University, Casablanca, Morocco; ^2^ Department of Agricultural Sciences, University of Naples Federico II, Naples, Italy

**Keywords:** phosphate-solubilizing *Streptomyces*, plant growth-promoting rhizobacteria, bioinoculant, biofertilizer, biostimulant, biocontrol

## Abstract

Currently, the use of phosphate (P) biofertilizers among many bioformulations has attracted a large amount of interest for sustainable agriculture. By acting as growth promoters, members of the *Streptomyces* genus can positively interact with plants. Several studies have shown the great potential of this bacterial group in supplementing P in a soluble, plant-available form by several mechanisms. Furthermore, some P-solubilizing *Streptomyces* (PSS) species are known as plant growth-promoting rhizobacteria that are able to promote plant growth through other means, such as increasing the availability of soil nutrients and producing a wide range of antibiotics, phytohormones, bioactive compounds, and secondary metabolites other than antimicrobial compounds. Therefore, the use of PSS with multiple plant growth-promoting activities as an alternative strategy appears to limit the negative impacts of chemical fertilizers in agricultural practices on environmental and human health, and the potential effects of these PSS on enhancing plant fitness and crop yields have been explored. However, compared with studies on the use of other gram-positive bacteria, studies on the use of *Streptomyces* as P solubilizers are still lacking, and their results are unclear. Although PSS have been reported as potential bioinoculants in both greenhouse and field experiments, no PSS-based biofertilizers have been commercialized to date. In this regard, this review provides an overview mainly of the P solubilization activity of *Streptomyces* species, including their use as P biofertilizers in competitive agronomic practices and the mechanisms through which they release P by solubilization/mineralization, for both increasing P use efficiency in the soil and plant growth. This review further highlights and discusses the beneficial association of PSS with plants in detail with the latest developments and research to expand the knowledge concerning the use of PSS as P biofertilizers for field applications by exploiting their numerous advantages in improving crop production to meet global food demands.

## Introduction

Phosphorus (P) is a macronutrient required in abundance by plants and is essential in many metabolic reactions, as P acts as a backbone in molecules such as deoxyribonucleic acid (DNA), ribonucleic acid (RNA) and phospholipids of plant cells, as well as it is essential in photosynthesis and improves flower formation, seed production, crop maturity and, resistance to plant diseases, stimulates root development and supports the plant development throughout entire life cycle (Soumare et al., 2020). Additionally, P is the second limiting nutrient after nitrogen in agricultural production. For this reason, most farmers regularly apply P chemical fertilizers to avoid P deficiency, which leads to stunted growth and dark-green coloration of plants, alters the plant metabolism by accumulating carbohydrates especially sucrose in leaves and secretion of organic acid (Meng et al., 2021). Typically, the challenge of soil phosphorus deficiency is addressed by the use of P-based fertilizers, which usually precipitate immediately after their application and tend to be unavailable to plants, thus wasting P (Urrutia et al., 2014). Often, the excess use of chemical P can lead to severe risks to environmental and human health, such as groundwater contamination and waterway eutrophication (Alori and Babalola, 2018). Beside their negative environmental impact, chemical P-based fertilizers are expensive due to their extraction costs from the raw materials (P rock) and the substantial amounts of energy required to transform them into forms that farmers can use (Abebe et al., 2022). This high cost could be balanced by investing money in an alternative procedure with a greater potential return. P-biofertilizers provides benefits from an economic, social, and environmental point of view since they can help to reduce the cost of P input because of their possibility to use the accumulated P in soils and enhance plant growth (Khan et al., 2007), as well as is need a smaller amount of energy for their production in respect to chemical fertilizers (Carvajal-Muñoz and Carmona García, 2012) and they do require only a specific set of equipment to ferment correctly microbial strains and store them until use (Malusá et al., 2012). Therefore, the development and use of bioformulations with beneficial microbes able to improve the growth, fitness and health of plants growing in soils lacking nutrients and/or experiencing stress is an attractive low-cost alternative to agrochemical management (Romano et al., 2020a). In particular, the use of phosphate-solubilizing microorganism (PSM)-based inocula is a promising ecofriendly strategy to increase soil P availability as well as to enhance P absorption by plants, thereby limiting the use of chemical fertilizers (Walpola and Yoon, 2012; Kishore et al., 2015). Among soil microbes, *Streptomyces* and other Actinomycetes are among the dominant prokaryotic taxa living in these environments. *Streptomyces* contains many species with different plant growth-promoting activities (Olanrewaju and Babalola, 2019; Di Mola et al., 2021); these activities affect a wide range of biological processes by increasing soil nutrient availability to plants and phytohormone production and/or by suppressing plant disease by inhibiting the growth of soil-borne plant pathogens (Bhatti et al., 2017). Specifically, several species with different morphological and functional characteristics are considered PSMs for their potential ability to release insoluble P and make it available for plants (Hamdali et al., 2010; Chouyia et al., 2020). Despite their several interesting biotechnological activities, *Streptomyces* species have been scarcely used as commercial microbe-based biofertilizer components.

Although the various implications and interactions in plant growth promotion by member of the genus *Streptomyces* have been largely reviewed (Amaresan et al., 2018; Olanrewaju and Babalola, 2019) and the use of *Streptomyces* species in agriculture has been recently reported (Rey and Dumas, 2017), very little is known about their ability to solubilize P. Thus, based on these considerations, this review gives insights into the current knowledge on the ability of *Streptomyces* species to solubilize P, with a focus on the mechanisms through which the solubilize and mineralize P. Moreover, this review also describes plant growth-promoting traits coupled to P solubilization and examines the potential of applying *Streptomyces* an innovative biofertilizer in crop production.

## Taxonomic characteristics, nature, and habitat of *Streptomyces*

*Streptomyces*, belonging to the order Streptomycetales, family Streptomycetaceae, are aerobic spore-forming filamentous gram-positive bacteria, which comprises 1131 species and 72 subspecies that are validly described (https://lpsn.dsmz.de/genus/streptomyces). They are distinguished from most other bacteria by their mode of growth and their properties of both fungal and bacterial cell morphologies (Das et al., 2008). *Streptomyces* colonies are slow growing and produce aerial mycelia responsible for the characteristic soil odor because of the production of the geosmin as volatile metabolite (Ikeda et al., 2003; Jüttner and Watson, 2007). Preferring a neutral or slightly alkaline pH, *Streptomyces* species are generally mesophilic, while others are thermophilic and tolerate temperatures of approximately 50°C and even up to 60°C (Shivlata and Satyanarayana, 2015). The genome of *Streptomyces* is characterized by large linear chromosomes with a high guanine–cytosine content, while the most marked characteristic of the *Streptomyces* genome is the high degree of chromosomal instability, which leads to frequent spontaneous deletions and rearrangements, especially at the ends of the chromosome (Lee et al., 2020). They are well known as dominant prokaryotic taxa that live in the soil and have been found in various habitats, including rivers, lakes, and marine ecosystems; this ubiquitousness is due to their ability to produce a large number of specific enzymes and secondary metabolites as well as their filamentous forms which cause the strength of soil texture and allow them to compete in various microbial communities and survive in various habitats, even in desert soils (Jensen et al., 2005; Radhakrishnan and Balagurunathan, 2011; Ventorino et al., 2016; Montella et al., 2017; Sheridan et al., 2017; Pennacchio et al., 2018; Ventorino et al., 2019). The size and the activity of the *Streptomyces* population increase in soil comparing to other bacteria because of their ability to decompose plant and animal residues, degrade cotton textiles, plastics, rubber, and paper as well as cellulose, lignocellulose, chitin, and different organic compounds to survive as they can recovered even from horizon C of the soil (Hasani et al., 2014). Based on those functions, *Streptomyces* influence their environment, e.g., soil biogeochemical cycles, soil community structure and/or other microorganisms (Bontemps et al., 2013; Donald et al., 2022). In a recent study, denaturing gradient gel electrophoresis (DGGE) analysis indicated that *S. roseocinereus* MS1B15 inoculation affected microbiota in the rhizosphere of barley plants by increasing prokaryotic and eukaryotic diversity comparing to un-inoculated soil (Chouyia et al., 2020). In addition, Wang et al. (2020) pointed that the application of the strain *S. lydicus* M01, which exhibited growth promoting characteristics such as P-solubilization, IAA secretion, siderophore and ACC deaminase production as well as increased cucumber plant growth, in addition to determining an increase in the abundance of *Streptomyces* genus altered the microbiota composition by promoting prokaryotic and eukaryotic populations known as potentially beneficial microorganisms, such as *Pseudarthrobacter*, *Sphingomonas*, *Rhodanobacter*, *Pseudomonas*, *Fusicolla*, *Humicola*, *Solicoccozyma*, and *Paraphaeosphaeria*.

## Phosphate-solubilizing *Streptomyces*

Phosphorus is considered an essential nonrenewable macronutrient required for the growth and development of plants. P nutrition is associated with several key functions of plants, including the development of roots, the strengthening of stems, the formation of flowers and seeds, plant maturity and quality of production (Walpola and Yoon, 2012). Although a substantial reserve of P is present in the soil, a large portion is unavailable to plants, and a considerable portion of phosphate applied to soils as fertilizer is rapidly immobilized, leading to low P-use efficiency and potentially excess P (Kochian, 2012). The solubilization and microbial mineralization of P is an ecofriendly and low-cost strategy to exploit native soil P, limiting the application of chemical fertilizers and providing both environmental and economic benefits (Zaidi et al., 2009). Microorganisms capable of solubilizing phosphate are called PSMs, which mainly include Actinomycetes. Hamdali et al. (2008a) reported that among 300 actinobacterial isolates, approximately 20% were able to efficiently solubilize P and belong to the *Streptomyces* genus. Numerous *Streptomyces* species isolated from rhizosphere soils have been reported as potential P solubilizers (Hamdali et al., 2010), and they are able to solubilize different sources of P; several works have reported the ability of *Streptomyces* to solubilize the tricalcium P form. In particular, different *Streptomyces* species isolated from wheat and tomato rhizospheres were shown to be able to produce high amounts of P, ranging from 70.36 µg/ml to 1916.12 ± 10.35 µg/ml (Jog et al., 2012; Sadeghi et al., 2012; Jog et al., 2014; Anwar et al., 2016; **Table 1**). By the use of a semiquantitative method, the strain *Streptomyces* sp. PSA-7, isolated from forest soil in the Mahabubnagar district, was shown to solubilize tricalcium P, producing a halo of 27 mm (Balakrishna et al., 2012). Similarly, the strains *Streptomyces violascens* TNC-1 and *Streptomyces asenjonii* MNC-1, isolated from extreme environments in Morocco, as well as *Streptomyces* sp. VITMS22, isolated from an agricultural field, were able to produce P starting from its tricalcium form, albeit at low amounts ranging from 8.56 µg/ml to 20 µg/ml (Kizhakedathil and Devi, 2018; Nafis et al., 2019). In contrast, Jog et al. (2014) reported that the strain *Streptomyces cellulose* mhcr 0816, isolated from a wheat rhizosphere, exhibited high P solubilization (up to 1916 mg/L), a value that exceeded the level of other well-known P solubilizing bacterial genera, such as *Bacillus* (up to 957 mg/L; Mehta et al., 2010) and *Pseudomonas* (up to 66.2 ± 13.4 mg/L; Blanco-Vargas et al., 2020), which were previously reported as potential PSMs (Sharma et al., 2013; Kalayu, 2019).

**Table 1:** Studies on the phosphate-solubilizing *Streptomyces* species within past 10 years.

Species	Source	P-solubilizing concentration (µg/ml)	P form	Location	References
*Streptomyces* sp. WA-1	Wheat and tomato fields	72.13	Ca_3_(PO_4_)_2_	Punjab province (District: Lahore, Gujranwala, Sheikhupura) Pakistan	Anwar et al. (2016)
*S. djakartensis* TB-4		70.36			
*S. rochei* IDWR19	Wheat rhizosphere	95.40 ± 5.1		Idar region of Gujarat, India (lat 23° 50′ E and long N 73° 02′)	Jog et al. (2012)
*S. carpinensis* IDWR53		405.17 ± 6.2			
*S. thermolilacinus* IDWR81		911.6 ± 5.3			
*S. cellulosae* mhcr 0816	Wheat rhizosphere	1916.12 ± 10.35		Mehsana region of Gujarat, India (latitude 23° 42′ N and longitude 72° 33′ E)	Jog et al. (2014)
*S. tricolor* mhce 0811		950.23 ± 9.20			
*Streptomyces* sp. C	Wheat soil	92		Kerman, Iran	Sadeghi et al. (2012)
*Streptomyces* sp. PSA-7	Forest soils of Mahabubnagar district	27 mm (semi quantitative measurement)		Nallamala forest of Mahabubnagar district of Andhra Pradesh, (Lat 160 221 3511 N, 780 451 2111E).	Balakrishna et al. (2012)
*S. violascens* TNC-1	Mountain soil	12.39		Morocco	Nafis et al. (2019)
*S. asenjonii* MNC-1	Desert soil	8.56			
*Streptomyces* sp. VITMS22	Agriculture field	20		Kolathur village, Tamil Nadu	Kizhakedathil and Devi (2018)
*S. alboviridis* P18	Desert soil	32	Rock phosphate	Morocco	Boubekri et al. (2021)
*S. griseorubens* BC3		30.9			
*S. griseorubens* BC10		31.2			
*S. fulvissimus* AN-12	Compost from villages of barshidist-solapur	152.86 ± 0.03	Ca_3_(PO_4_)_2_	Barshi Dist-Solapur, MS, India	Nandimath et al. (2017)
		144.59 ± 0.04	Rock phosphate		
*S. olivoverticillatum* AN-20		152.86 ± 0.03	Ca_3_(PO_4_)_2_		
		180.87 ± 0.07	Rock phosphate		
*S. nogalater* AN-24		102.69 ± 0.02	Ca_3_(PO_4_)_2_		
		109.62 ± 0.03	Rock phosphate		
*S. longisporoflavus* AN-27		191.25 ± 0.02	Ca_3_(PO_4_)_2_		
		190.36 ± 0.04	Rock phosphate		
*S. cellulosae* AN-31		172.72 ± 0.04	Ca_3_(PO_4_)_2_		
		145.33 ± 0.05	Rock phosphate		
*Streptomyces* sp. L3	Rhizosphere	309.85 ± 8.78	Ca_3_(PO_4_)_2_	–	Chaiharn et al. (2018)
		198.96 ± 5.19	Rock phosphate		
		15.37 ± 1.78	Alpo_4_		
*Streptomyces* sp. KT 6-4-1		310.00 ± 6.32	Ca_3_(PO_4_)_2_		
		250.35 ± 5.43	Rock phosphate		
		37.59 ± 4.37	AlPO_4_		
*Streptomyces* sp. ST 3		300.75 ± 9.83	Ca_3_(PO_4_)_2_		
		245.34 ± 3.11	Rock phosphate		
		30.98 ± 3.11	AlPO_4_		
*S. roseocinereus* MS1B15	Oats rhizosphere	245.6 ± 11	CaHPO_4_	Northwest of Morocco (33°32′ 00″N, 7°35′00″W)	Chouyia et al. (2020)
*S. rochei WZS1-1*	Rhizosphere of *mikaniamicrantha*kunth	28.49 ± 0.30	Organic phosphate	Qiongzhong, Hainan (N19°01’40”, E109°41’37”)	Han et al. (2018)
*S. sundarbansensis WZS2-1*		5.52 ± 0.41			
*S. alboniger* and *S. venezuelae* 43	Soil ecosystems (forest, pasture, rain fed and irrigated cultivated land)	1.116		–	Ghorbani-Nasrabadi et al. (2012)
*S. ambofaciens* and *S. lienomycini* 63		0.435			
*Streptomyces* sp. KP109810	–	562	Single super phosphate	–	Mohammed (2020)
*S. rishiriensis* 3AS4	Wheat rhizosphere	400	Phytate	Palmital (S 22° 47′ 30″; W 50° 12′ 18″) São Paulo State, and Planaltina (S 15° 36′; W 47° 42′) Brasília.	Vargas Hoyos et al. (2021)
		300	Ca_3_(PO_4_)_2_		
		200	Rock phosphate		

In addition to their ability to solubilize tricalcium P, *Streptomyces* spp. are also able to solubilize other inorganic P forms, such as rock P (Nandimath et al., 2017; Boubekri et al., 2021), aluminum P (Chaiharn et al., 2018), and dicalcium P (Chouyia et al., 2020). However, *Streptomyces* species are also able to mineralize organic P (Han et al., 2018), as reported also Ghorbani-Nasrabadi et al. (2012); specifically, *Streptomyces alboniger, Streptomyces venezuelae* 43, *Streptomyces ambofaciens* and *Streptomyces lienomycini* 63, isolated from different soil ecosystems, were shown to solubilize both tricalcium and organic P by producing phytase enzymes (**Table 1**). Nevertheless, the strain *Streptomyces rishiriensis* 3AS4, isolated from a wheat rhizosphere cultivated in the Cerrado biome, was found to use several P forms. In detail, this strain was able to mineralize and solubilize phytate as well as tricalcium and rock P, reaching final concentrations of 400 μg/ml, 300 μg/ml and 200 μg/ml, respectively (Vargas Hoyos et al., 2021; **Table 1**).

## Mechanism of P solubilization by *Streptomyces*

**Figure 1:**
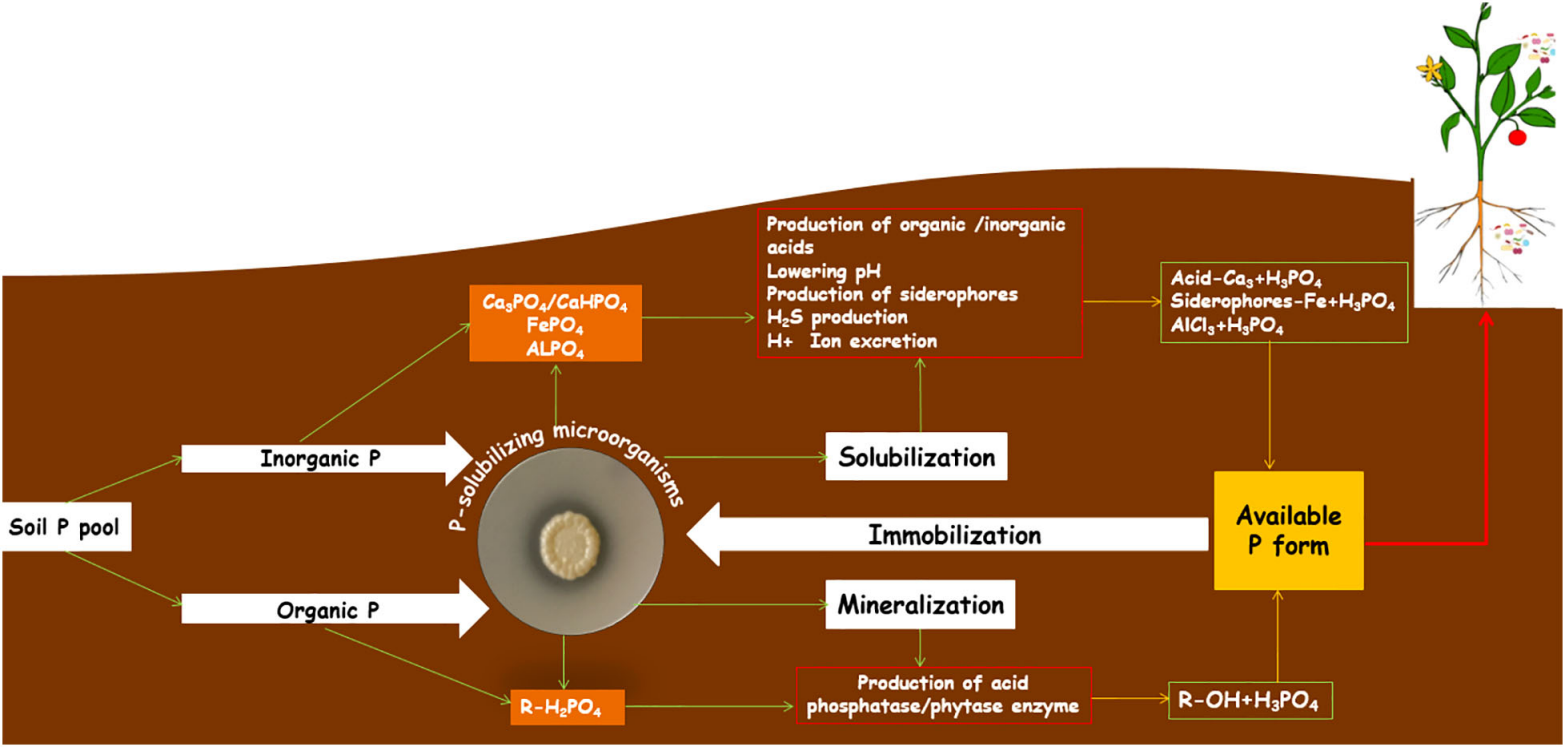
Schematic representation of solubilization/mineralization mechanisms of different P forms by P-solubilizing *Streptomyces*. Soil P is present in inorganic and organic forms. Phosphate-solubilizing *Streptomyces* induce P reactions by several mechanisms (production of organic and inorganic acids, siderophores, etc.) to release unavailable and immobilized inorganic P forms for plants (Ca-P, calcium phosphate; Fe-P, iron phosphate; Al-P, aluminum phosphate) by solubilization; while they secret extracellular enzymes (phosphatase and phytase) to release the organic P forms (orthophosphate, phosphonates, etc.) by mineralization process in order to provide the water-soluble form H_3_PO_4_ to the plant.

The chemical P added to the soil by farmers to avoid P-limiting conditions in cropping systems usually precipitates after the application by the formation of unavailable P complexes, whether in acidic or alkaline soils (Urrutia et al., 2014). This mechanism generally results in a slow release of P, causing great challenges for remediation of these soils, with a high level of P unavailable to plants (Roy, 2017); plants prefer P in a water-soluble form, i.e., PO_4_^3−^, H_2_PO_4_^2−^, HPO_4_^2−^. Concentrations of these water-soluble P forms are very low in soils and vary from 0.001 mg/L (poor soils) to 1 mg/L (highly fertile soils). PSMs are known to transform inorganic and organic P sources to soluble forms and make them available to plants by solubilization (Toro, 2007; Walpola and Yoon, 2012) and/or by mineralization of complex P compounds (Glick, 2012). There are several hypotheses for describing the mechanisms of P solubilization by microbes (Khan et al., 2007; Buch et al., 2008). **Figure 1** shows a schematic representation of the solubilization/mineralization of P forms by PSS. First, the excretion of organic acids has been widely accepted as the main mechanism of inorganic P solubilization, and various studies have pointed out the role of organic acid production in the solubilization process (Antoun, 2012; Marra et al., 2012; Alori and Babalola, 2018). The organic acids produced by PSMs in nature or under *in vitro* conditions act through i) reducing the pH, ii) enhancing the chelation of cations bound to P, iii) forming complexes with P-associated metal ions and iv) competing with P for adsorption (Pradhan and Sukla, 2006; Ingle and Padole, 2017). Consequently, the excretion of these organic acids is accompanied by acidification of the microbial cells and their surroundings; therefore, P ions are released by H^+^ being substituted for Ca^2+^ (Jones and Oburger, 2011). Hence, Sharan and Darmwal (2008) proposed that assimilation of NH_4_^+^ together with H^+^ elicits about P solubilization. Indeed, Park et al. (2009) confirmed that for some microorganisms, NH_4_^+^-driven H^+^ release seems to be the sole mechanism to promote P solubilization.

The excretion of inorganic acids, e.g., HCl, H_2_SO_4_ and HNO_3_, has also been reported to lead to the solubilization of P (Reyes et al., 2001; Richardson, 2001). The release of bound inorganic P by the production of H_2_S is another mechanism through which ferric phosphate is involved, resulting in the formation of ferrous sulfate and P release. This could be because of microbial S oxidation, nitrate production and CO_2_ formation, which lead to the formation of inorganic acids such as H_2_SO_4_. Jones and Oburger (2011) suggested that P microbial solubilization might also occur in response to the excretion of chelating substances such as siderophores that form stable complexes with P adsorbents. Interestingly, mineralization and immobilization of soil organic P play a key role in the phosphorus cycling of agricultural lands. PSMs also play an important role in mineralizing organic P forms and making organic P available to plants principally through the production of acid phosphatases (Beech et al., 2001; Kishore et al., 2015). Richardson and Simpson (2011) documented that half of the soil microorganisms and plant roots have P mineralization potential through the action of phosphatases. In the case of P solubilization by *Streptomyces*, several researchers have reported that this genus involves acidification of the growth medium and production of organic acids by the release of P (Arunachalam Palaniyandi et al., 2013; Farhat et al., 2015; Biglari et al., 2016). Recently, Chaiharn et al. (2018) indicated that numerous *Streptomyces* species were able to solubilize P by resulting in a significant pH decrease ranging from culture growth or the production of organic acids but with the secretion of chelating siderophore-like molecules.

Moreover, several *Streptomyces* species demonstrated the ability to mineralize organic P by the production of phosphoric acid. Walpola and Yoon (2012) documented that members of the genera *Bacillus* and *Streptomyces* can mineralize complex organic phosphates through the production of extracellular enzymes such as phosphoesterases, phosphodiesterases, phytases, and phospholipases. Additionally, Khan et al. (2009) reported that the ability of *Bacillus* and *Streptomyces* species to mineralize organic P was higher than that exhibited by *Aspergillus*, *Penicillium*, *Proteus, Serratia, Pseudomonas* and *Micrococcus* species, although the mixed PSM cultures (*Bacillus*, *Streptomyces*, *Pseudomonas*, etc.) were the most effective at mineralizing organic phosphate.

## Plant growth-promoting traits by phosphate-solubilizing *Streptomyces*

Beneficial soil bacteria such as PSS, which have been shown to make mainly soluble P accessible to plants in different production systems, are usually referred to as plant growth-promoting *Streptomyces* (PGPS) (Amaresan et al., 2018). In addition, by providing soluble P to plants, *Streptomyces* species exhibit a range of traits that promote plant growth, such as N_2_ fixation and the production of both growth-promoting hormones and many secondary metabolites. These beneficial attributes can be divided into those associated with biofertilization, biostimulation, and bioprotection.

### Phosphate-solubilizing *Streptomyces* as biofertilizers

Biofertilizers are organic in nature and include secondary metabolites of microbial origin or microorganisms themselves (Mishra and Dash, 2014). Thus, living microorganisms that directly affect processes that improve macro- and micronutrient acquisition, such as nitrogen fixation, P solubilization or nutrient mobilization, to increase soil or plant productivity can be used as biofertilizers. In addition to their ability to solubilize P, *Streptomyces* members can also facilitate the growth and development of plants by producing essential nutrients. Sellstedt and Richau (2013) pointed out that gram-positive actinobacteria, including species of *Streptomyces*, had nitrogen-fixing ability. Dahal et al. (2017) considered *Streptomyces* species that have the nitrogen fixation-related gene *nif*H to be a nitrogen-fixing bacteria, and as N-fixers essential for the functioning of soil ecosystem (Ventorino et al., 2007). On the other hand, *Streptomyces thermoautotrophicus* demonstrated the ability to fix atmospheric nitrogen into biological form by molybdenum denitrogenase and manganese-superoxide oxidoreductase (Ribbe et al., 1997).

Controversially, Mackellar et al. (2016) reported that *S. thermoautotrophicus* is a highly effective nitrogen scavenger but not a nitrogen fixer. Chouyia et al. (2020) established that, like PSS, the P-solubilizing strain *Streptomyces roseocinereus* MS1B15 isolated from the Moroccan oat rhizosphere did not contain *nifH* genes and therefore was considered a P-solubilizer with negative nitrogen fixation activity. By evaluating their growth on N-free media, Fatmawati et al. (2019) and Wahyudi et al. (2019) demonstrated that the PSS species isolated from a soybean rhizosphere were able to fix nitrogen (**Table 2**). To date, there is no convincing evidence for free-living and endophytic *Streptomyces* that can fix nitrogen, and their mechanism remains unclear.

**Table 2:** Studies on the plant growth promoting traits released by P-solubilizing *Streptomyces* species within past 10 years.

P-solubilizing *Streptomyces*	Source	Beneficial attributes	Plant growth promoting traits	Elicited effects	References
*S. roseocinereus* MS1B15	Oat rhizosphere	Biofertlizer, biostmulant and biocontrol	Siderophores, IAA, ACC deaminase and antimicrobial activity.	Increased shoot and ear length as well as available phosphorus in ears and leaves, P and N contents in the soil.	Chouyia et al. (2022)
*S. bellus*ASR 46 *S. tendae*ASR 58 *S. thermocarboxydus* ASR 75 *S. ramulosus* ASR 76	Soybean rhizosphere		Nitrogen fixation, IAA, siderophores, and chitinase	Increased soybean sprouts growth.	Fatmawati et al. (2019)
*S. panaciradicis* ARK 13 *S. recifensis* ARK 63 *S. polychromogenes* ARK 86 *S. manipurensis* ARK 94 *Streptomyces* sp. ARK 116			Nitrogen fixation, and IAA,	Promoted soybean growth by increasing hypocotyl and radicular length as well as the number of lateral roots.	Wahyudi et al. (2019)
*S. alboviridis* P18, *S. griseorubens* BC3, *S. griseorubens* BC10	Desert soil		K-solubilization, IAA, siderophore, HCN, and ammonia production	Improved the growth parameters of wheat.	Boubekri et al. (2021)
*S. violascens* TNC-1	Mountain soil		K-solubilization, IAA, nitrogen fixation activity, and siderophores	–	Nafis et al. (2019)
*S. asenjonii* MNC-1	Desert soil			–	
*S. bellus* AYD *S. enissocaesilis* BYC *S. tunisiensis* AI	Sugar beets		K –solubilization, IAA, antifungal activity	–	Aallam et al., 2021a
*S. rochei* IDWR19, *S. thermolilacinus* IDRWR81	Wheat rhizosphere		Siderophores, IAA, chitinase and cellulose.	Increased shoot length and biomass of wheat plant.	Jog et al. (2012)
*S. carpinensis* IDWR53				–	
*S. enissocaesilis*	Sugar beets	Biofertilizer	Siderophores	–	Aallam et al., 2021b
*Streptomyces* sp. WA-1	Wheat and tomato field	Biofertlizer, andbiostimulant	Siderophore, ammonia, and hydrogen cyanide production, IAA, ACC deaminase	Increased wheat root number	Anwar et al. (2016)
*S. djakartensis* TB-4				Increased wheat growth (root length, shoot length, plant fresh and dry weight, and number of leaves).	
*S. diastaticus* SP2	Healty medicinalplants		IAA, siderophores, chitinase and antifungal activity	Increased the biomass and reduced plant mortality of chickpea.	Singh and Gaur (2016)
*S. ossamyceticus* SP10					
*S. griseus* SP12					
P-solubilizing *Streptomyces*	Source	Beneficial attributes	Plant growth promoting traits	Elicited effects	References
*S. jiujiangensis* 2SH3-07	Water and sediment samples of mangrove areas	nbsp	Siderophores, IAA, antagonistic activity	Exhibited high PGP activities even up to 300 mm of NaCl stress. Enhanced shoot and root weight and root length of rice seedling under non-saline and up to 200 mm NaCl conditions.	Suksaard et al. (2017)
*S. psammoticus* 3SH5-05			Siderophores, IAA, ACC deaminase, antagonistic activity		
*S. werraensis *(S4) *Streptomyces *spp. (S12) *S. indiaensis *(R11)	Maize plantations		IAA, ACC, siderophores and ammonia,	–	Chukwuneme et al. (2020)
*S. pseudovenezuelae *(S20)				Improve Maize growth and drought stress at three soil moisture	
*Streptomyces* sp. SR13−2 (*S. rimosus /S. cellulosae*)	Rice rhizospheric soil		IAA	Enhanced rice shoot height, root lengths, and dry weight of shoot and root	Detraksa (2020)
*Streptomyces* sp. VITMS22	Agriculture field		IAA, HCN, siderophores, and ammonia production.	Increased plant growth of mustard	Kizhakedathil and Devi (2018)
*S. griseorubiginosus* YRA064 *S. lanatus* YRA079 *S. lavendulae* YRA088 *S. vinaceus* YRA115	rhizosphere of yam plants		IAA, antimicrobial activity	Promoted the growth of Arabidopsis seedlings.	Arunachalam Palaniyandi et al. (2013)
*Streptomyces* sp. BPSAC34	Medicinal plants	Biofertlizer, Biostimulant, and Biocontrol	IAA, Ammonia, siderophores HCN, chitinase and antifungal activity	Increased root and shoot height of chili plant	Passari et al. (2015)
*S. mutabilis* BPSAC42 *Streptomyces* sp. BPSAC2			IAA, ammonia, chitinase and antifungal activity	–	
*S. staurosporininus* Z.55.4.3 *S. eurocidicus* Z.56.2.2	Rhizosphere of olive trees		IAA, siderophores, ammonia, antibacterial and antifungal activity	–	Dede et al. (2020)
*Streptomyces* sp. DH32	–	Biostimulant, and Biocontrol	Cellulose, protease, amylase, antifungal	–	Kaur et al. (2013)
*Streptomyces* sp. ETR1	–			–	
*Streptomyces* sp. DH33	–		IAA, cellulose, protease, amylase, antifungal	–	
*Streptomyces* sp. MR9	–		Cellulose, amylase, antifungal	–	
*Streptomyces* sp. P2-3	–		IAA, ammonia, nitrogenfixation, chitinase, cellulase, amylase, antifungal	–	

Indeed, potassium (K), together with nitrogen and phosphorus, is among the essential elements needed for plant growth (Amtmann and Armengaud, 2007; Chen et al., 2008). It is known that potassium-solubilizing bacteria (KSB) can solubilize K-bearing minerals and convert insoluble K to soluble forms available for plant uptake (Etesami et al., 2017). Several studies have reported the potential effect of KSB as biofertilizers for agricultural improvement by reducing the use of agrochemicals and supporting ecofriendly crop production (Archana et al., 2013; Prajapati et al., 2013). *Streptomyces* strains have been found to release potassium-bearing rock powder by solid-state fermentation (Liu et al., 2016). Recently, Boubekri et al. (2021) reported that 30% of *Streptomyces* isolated from a Moroccan desert soil were able to solubilize both K and P and could be promising candidates for the implementation of efficient biofertilization strategies to improve soil fertility and plant yield under rock P and rock K fertilization. Indeed, *S. violascens* TNC-1 and *S. asenjonii* MNC-1 isolated from montane and desert soils, respectively, were reported to solubilize inorganic K, as they produced a clear halo zone on Aleksandrov agar (Nafis et al., 2019), and several PSS strains isolated from sugar beet plants were found to be able to release soluble K from insoluble orthoclase in large amounts ranging from 3.8 mg/L to 216.6 mg/L (Aallam et al., 2021a; **Table 2**).

In addition, iron (Fe) is an essential trace element required by all organisms. However, the oxidation state of iron can reversibly change, causing the ion to become inaccessible by microorganisms. The production of siderophores is one strategy employed by microorganisms to survive in iron-stressed environments and overcome low iron availability (Haas, 2003; Ma, 2005). Imbert et al. (1995) reported that different *Streptomyces* species were able to produce different types of siderophores. In particular, *Streptomyces viridosporus* produced both linear desferrioxamine B and cyclic desferrioxamine E, while *S. ambofaciens* synthesized only cyclic desferrioxamine E as the main siderophore; finally, linear desferrioxamine G was the major form produced by *Streptomyces coelicolor* and *Streptomyces lividans*. *Streptomyces* sp. GMKU 3100 isolated from the roots of a Thai jasmine rice (*Oryza sativa* L. cv. KDML105) plant and selected as the highest siderophore producer on CAS agar media, significantly promoted rice and mungbean plant growth in a greenhouse experiment and therefore could be applied as a potentially safe and environmentally friendly biofertilizer in agriculture (Rungin et al., 2012). The strains *Streptomyces rochei*, *Streptomyces carpinensis* and *Streptomyces thermolilacinus* isolated from a wheat rhizosphere as well as *Streptomyces enissocaesilis* isolated from sugar beet selected as the best P solubilizers were also capable of producing siderophores on CAS agar media (Jog et al., 2012; Aallam et al., 2021b; **Table 2**). Similarly, several works have demonstrated the ability of numerous PSS strains isolated from different sources to produce siderophores in addition to their main P activity (Anwar et al., 2016; Suksaard et al., 2017; Singh et al., 2019; **Table 2**).

### Phosphate-solubilizing *Streptomyces* as biostimulants

PGPS can regulate plant growth by the production and/or degradation of plant hormones composing major groups (Duncan et al., 2015). Phytohormones, such as auxins, cytokinins (CKs), gibberellin (GA), and ethylene (ET), are important growth regulators synthesized in defined organs of plants and have a prominent impact on plant metabolism (Fahad et al., 2015; Shi et al., 2017). Numerous *Streptomyces* strains, such as *Streptomyces* CMU-PA101, *Streptomyces* CMU-SK126 (Khamna et al., 2009), *Streptomyces atrovirens* ASU14 (Abd-Alla et al., 2013) and *Streptomyces fradiae* NKZ-259 (Myo et al., 2019), have been extensively reported to produce the auxin indole-3-acetic acid (IAA). Similarly, Rashad et al. (2015) reported the ability of *Streptomyces* strains isolated from a marine environment to produce a wide range of phytohormones, including GA, IAA, abscisic acid, kinetin and benzyladenine, and were able to positively influence the growth parameters of eggplant (*Solanum melongena*), including root length and fresh/dry root weight, in pot experiments. Furthermore, numerous *Streptomyces* strains, in addition to their main P-solubilizing activity, were able to secrete IAA in broth media, reaching values from 7.9 ± 0.1 µg/ml to 122.3 ± 0.1 µg/ml (Aallam et al., 2021a; **Table 2**). The P-solubilizing, *Streptomyces griseorubiginosus* YRA064, *Streptomyces lanatus* YRA079, *Streptomyces lavendulae* YRA088, *Streptomyces vinaceus* YRA115, isolated from the rhizosphere of yam plants, *Streptomyces* sp. SR13−2, isolated from a rice rhizosphere, *Streptomyces pseudovenezuelae* (S20), isolated from a maize plantation, and *Streptomyces* sp. VITMS22, isolated from an agricultural field, were reported to secrete IAA and enhance biometric parameters of Arabidopsis seedlings, rice plants, maize plants, and mustard plants (Arunachalam Palaniyandi et al., 2013; Kizhakedathil and Devi, 2018; Chukwuneme et al., 2020; Detraksa, 2020; **Table 2**).

*Streptomyces* strains also degrade 1-aminocyclopropane-1-carboxylate (ACC). ACC deaminase-producing *Streptomyces filipinensis* No. 15 and *S. atrovirens* No. 26 were shown to increase plant growth significantly through the reduction of levels of endogenous ACC and the consequent lowering of endogenous ET levels in plant tissues (El-Tarabily, 2008). P-solubilizing *Streptomyces nobilis* WA-3 was selected as the most active IAA producer by Anwar et al. (2016); this strain showed a significant ability to produce ACC deaminase. Due to its ability, this strain significantly increased several growth parameters of wheat plants in pot experiments under standard conditions (**Table 2**).

### Phosphate-solubilizing *Streptomyces* as biocontrol agents

*Streptomyces* has also been investigated for the production of bioactive molecules with an antagonistic function against plant pathogens (Barakate et al., 2002; Toumatia et al., 2015). The main biocontrol ability of *Streptomyces* species is attributed to their high production of antibiotics and hydrolytic enzymes such as cellulase, amylase, protease, and xylanase (Dhanasekaran et al., 2008; Al-Askar et al., 2015). *Streptomyces griseoviridis*, which was isolated from a tomato rhizosphere soil, was shown not only to exhibit antagonistic activity against *Fusarium* spp., *Botrytis* spp., and *Alternaria* spp. but also to exert potential antagonistic activity against *Rhizoctonia solani*, *Pythium* spp., *Phytophthora* spp., *Thielaviopsis basicola*, and *Verticillium dahlia*, demonstrating the wide-ranging potential of the members of this genus (Sabaratnam and Traquair, 2015). In addition, several *Streptomyces* strains, such as *Streptomyces hygroscopicus, Streptomyces malaysiensis*, *Streptomyces violaceusniger* YH27A, *S. coelicolor*, *S. violaceusniger* YCED-9, and *Streptomyces* sp. isolate KY-33, isolated from rhizosphere soil, have been reported to be potential antifungal agents against different soil-borne plant pathogens (Cheng et al., 2010; Gherbawy et al., 2012; Wu et al., 2012; Ghadge and Patil, 2016; Som et al., 2017; Shrivastava and Kumar, 2018).

The P-solubilizing *Streptomyces griseus* BH7 strain, isolated from Moroccan phosphate mines, was shown to inhibit the growth of potentially phytopathogenic fungi, bacteria (gram+/-) and yeasts. Therefore, this strain could be used for the development of novel biofertilizer and biocontrol products composed of spores and/or mycelia of the *ad hoc* Actinobacteria in association with pulverized rock P (Hamdali et al., 2008b).

Furthermore, the strains *Streptomyces* sp. BPSAC34, *Streptomyces mutabilis* BPSAC42, and *Streptomyces* sp. BPSAC2, isolated from medicinal plants, demonstrated potential to solubilize inorganic P for sustainable agriculture, as these strains positively affected multiple plant growth traits as well as exerted antifungal activity against fungal phytopathogens (Passari et al., 2015). The P-solubilizing strain *S. griseus* SP12 demonstrated a strong inhibitory effect—more than 60%—against *Sclerotium rolfsii, R. solani*, and *F. oxysporum*, reducing chickpea mortality (Singh and Gaur, 2016; **Table 2**), while Suksaard et al. (2017) reported that, in addition to their strong ability to solubilize P under various saline conditions, the P-solubilizing strains *Streptomyces jiujiangensis* 2SH3-07 and *Streptomyces psammoticus* 3SH5-05 have potential antagonistic activity against the rice pathogenic bacterial strains *Xanthomonas oryzae* pv. *oryzae* and *X. oryzae* pv. *oryzicola*. Other P-solubilizing actinobacterial strains also exert antimicrobial activity against bacteria. In particular, *Streptomyces eurocidicus* Z.56.2.2 and *Streptomyces staurosporininus* Z.55.4.3 showed very high inhibitory activity against several pathogenic bacteria and fungi, including *Pseudomonas* pv. *glycinea, Staphylococcus aureus, Enterococcus faecalis, Aspergillus parasiticus*, and *Aspergillus niger* and against *S. aureus Phaseolus vulgaris*, respectively (Dede et al., 2020). In some cases, the antimicrobial activity of several PSS species was associated with the hydrolytic activity of specific enzymes, such as cellulose, protease, and amylase, as reported by Kaur et al. (2013) (**Table 2**).

## Development and applications of phosphate-solubilizing *Streptomyces* based biofertlizers

The use of PSMs as microbial inoculants in the soil appears to be an attractive, ecofriendly, and low-cost alternative for meeting agricultural challenges. In fact, the PSM strategy provides an excellent opportunity to satisfy global food demands, maintain a high quality of agricultural production, and limit both yield losses and the use of chemical fertilizers (Figueiredo et al., 2010; Fiorentino et al., 2018; Romano et al., 2020b). Many works have reported the potential biotechnological use of *Streptomyces* in agriculture. *Streptomyces griseoflavus* was tested in combination with *Bradyrhizobium* strains as a biofertlizer in pot experiments, and these strains demonstrated a significant increase in plant growth; nodulation; nitrogen fixation; seed yield; and nitrogen, phosphorus and potassium (NPK) uptake and in mung bean and soybean (Htwe et al., 2019). Doolotkeldieva et al. (2015) reported a strong stimulatory effect of *Streptomyces fumanus*-based biofertilizer on the growth of seeds and seedlings of wheat and soybeans; this biofertilizer improved the composition of the rhizosphere microflora, attracted saprophytic microorganisms as ammonifiers and oligotrophs and promoted the growth and development of useful groups of nitrogen-fixing bacteria. Additionally, thermotolerant P-solubilizing *Streptomyces fulvissimus* AN-12, *Streptomyces olivoverticillatum* AN-20, *Streptomyces nogalater* AN-24, *Streptomyces longisporoflavus* AN-27 and *Streptomyces cellulosae* AN-31 have also been reported to be potential biofertilizers; these strains can be added as raw materials to agricultural waste to make the biofertilizer multifunctional and provide the ability to solubilize P and degrade all types of wastes, such as cellulose, carbohydrate, lipids, chitin and pectin, all of which represent the main macromolecular components of agricultural waste (Nandimath et al., 2017). Although several studies have reported the potential use of *Streptomyces* species as microbial inoculants in soils, only a few *Streptomyces* strains, such as *S. griseoviridis* and *Streptomyces lydicus*, have been developed commercially as biofertilizers to promote plant growth (Hamedi and Mohammadipanah, 2015). In fact, the development and production of a successful biofertilizer based on beneficial microorganisms such as phosphate-solubilizing *Streptomyces* needs a long process to supply reliable and contaminant-free bioproducts to the farmers. The full process usually includes i) preparation of inoculum by evaluating its properties on a proper medium to optimize growth parameters (Zhang et al., 2019), ii) selection of specific method of propagation, pilot-scale study, large-scale production, and quality testing at each level to ensure the success of fermentation process and to produce enough amounts of microbial cells (Trujillo-Roldán et al., 2013), iii) selection of the best carrier which can help to keep the microbes in good physiological conditions and provide better shelf life to biofertilizer formulation during the storage and in the application site, iv) evaluation of bioformulation efficiency by greenhouse and field experiments as thoroughly established by Ahmad et al. (2018), testing different environmental conditions and different plant species (Bashan et al., 2014), v) and suitable packaging reporting recommendation for application and the expiration date (Bhattacharjee and Dey, 2014). In this context, PSS have been applied as potential inoculants for many crop plant species under pot and field conditions, some of which are previously presented in **Table 2**. **Table 3** lists the beneficial effects exhibited by PSS species on different crop plants under greenhouse and natural conditions. Chaiharn et al. (2018) and Suárez-Moreno et al. (2019) reported that PSS species enhanced biometric parameters of rice plants under greenhouse and gnotobiotic conditions through P-solubilization activity as well as through promoting other plant growth-promoting traits (**Table 3**). In a study of wheat plants in a greenhouse experiment conducted by Sadeghi et al. (2012), *Streptomyces* sp. C significantly increased the germination rate, percentage and uniformity; shoot length; and dry weight, as well as the concentrations of N, P, Fe and Mn in the shoots. A similar study was conducted on wheat plants using *Streptomyces tricolor* mhce 0811, which was able to induce strong P-solubilizing activity that significantly increased wheat plant growth and mineral nutrient (Fe, Mn, and P) contents in the plants (Jog et al., 2014). The P-solubilizing strains *S. rochei WZS1-1*, *Streptomyces sundarbansensis WZS2-1* and *S. rochei* UU07 significantly increased several biometric parameters of wheat plants (Han et al., 2018; Singh et al., 2019; **Table 3**). A greenhouse experiment was conducted on chickpea plants involving the use of *S. griseus* SP12, which caused a significant increase in several plant biometric parameters (Singh and Gaur, 2016). Similarly, maize (*Zea mays* L.) plants inoculated with *Streptomyces* sp. KP109810 and cultivated in a greenhouse presented significant increases in growth and P nutrition (Mohammed, 2020), while soybean plant height as well as shoot and root volumes significantly increased in response to *S. rishiriensis* 3AS4 inoculation under glasshouse conditions (Vargas Hoyos et al., 2021). In addition, owing to their P-solubilizing activity and production of auxin, numerous *Streptomyces* species were shown to act as plant promoters of tomato (*Solanum lycopersicum*) plants (Djebaili et al., 2020). A more recent study by Chouyia et al. (2022) reported that, compared with a popular commercial biofertilizer containing arbuscular mycorrhizal fungi, the strain *S. roseocinereus* MS1B15, which has multiple plant growth-promoting activities but was selected mainly for its strong P-solubilizing ability, significantly enhanced tomato growth as well as yield and quality under different crop rotation schemes, indicating that this strain could represent a sustainable and feasible model system for tomato production and thus could be a potential biofertilizer candidate to increase plant growth as well as P uptake.

**Table 3:** Effect of PSS on growth and yield performance of different crops within past 10 years.

P-solubilizing *Streptomyces*	Host plant	Growth conditions	Effect on plant growth promotion	References
*Streptomyces* sp. KT 6-4-1 *Streptomyces* sp. ST 4-2-1	Rice plant	Greenhouse	Increased plant height and plant dry weight	Chaiharn et al. (2018)
*Streptomyces* A20 *Streptomyces* 5.1 *Streptomyces* 7.1	Rice (Fedearroz 733 and Fedearroz 60)	Gnotobiotic and greenhouse experiments	Improved growth of rice	Suárez-Moreno et al. (2019)
*Streptomyces* sp. C	Wheat	Greenhouse	Increased germination rate, percentage and uniformity, shoot length and dry weight, the concentration of N, P, Fe and Mn in wheat shoots	Sadeghi et al. (2012)
*S. tricolor* mhce 0811	Wheat *(Triticum aestivum* L.*)*	Plant growth chamber (milestone)	Improved plant growth, biomass and mineral (Fe, Mn, P) content	Jog et al. (2014)
*S. rochei WZS1-1* *S. sundarbansensis WZS2-1*	Wheat	In plant growth room (25/18 ± 2 °C (day/night) for 4 days)	Promoted wheat plant	Han et al. (2018)
*S. rochei* UU07	Wheat	Petri plate at room temperature	Increased root and shoot length, roots number and fresh weight biomass	Singh et al. (2019)
*S. griseus* sp12	Chickepea	Greenhouse	Increased shoot, root length, and fresh, dry weight of plant biomass.	Singh and Gaur (2016)
*Streptomyces* sp. KP109810	Maize *(Zea mays* L.)	Greenhouse	Improved crop growth and P nutrition	Mohammed (2020)
*S. rishiriensis* 3AS4	Soybean	Glasshouse	Increased plant height, the shoot and roots volume of soybean	Vargas Hoyos et al. (2021)
*S. xantholiticus* G33 *S. albidoflavus* H12 *S. thinghirensis* J4, K23 *S. anulatus* J13 *S. ambofaciens* J27	Tomato (*Solanum lycopersicum* L.)	Greenhouse	Promoted tomato plant growth by increasing length and dry weight of shoot and roots	Djebaili et al. (2020)
*S. roseocinereus* MS1B15	Tomato	Greenhouse	Increased tomato growth and yield, nutrient assimilation, and vitamin C under different crop rotation.	Chouyia et al. (2022)

The most reported PSS have been used as bioinoculants for sustainable agriculture not only for their P-solubilizing activity but also for their multiple functionalities to benefit plants by improving nutrient contents and inhibiting the growth of plant-borne pathogens. However, extensive research and more field trials are needed to study the mechanisms of P solubilization by *Streptomyces* and to create and develop a new commercial PSS-based biofertilizer.

## Challenges for developing phosphate-solubilizing *Streptomyces-*based biofertilizer

Numerous bio-inoculants do not show the same efficient potential after having been commercialized, presenting extremely bad quality and totally unreliable under field conditions. The main challenges in the industrial development of biofertilizers are often related to the performance of microbial inoculum and the quality control of the products at each stage of the process. In fact, during the process should be necessary to evaluate physiological parameters such as the medium composition, temperature, pH, moisture content, aeration, and agitation as well as identify produced cells by gram reaction and microscope observation and perform viable counting to ensure the purity and high quantity of cells (Herrmann and Lesueur, 2013).

The inadequate formulation is another bottleneck for the commercialization, which requires a particular suitable carrier to keep the microbial cells alive during the storage and transport. A good carrier should be: (i) nontoxic to organisms (ii) easily pulverized and sterilized (iii) able to carry exceptionally high number of microbial cells (iv) easily available and cost effective, and (v) should have a good absorption capacity (Mishra and Arora, 2016). Finally, bioformulates should have a long shelf life and stability.

The abiotic (e.g., soil type, soil characteristics, water content, environmental conditions and agriculture practices) and biotic factors (e.g., plant varieties and genetics and soil microbiome) and the mode of application, can be a critical issue to the biofertilizer efficiency (Newitt et al., 2019). However, the development of PSS *Streptomyces*-based biofertilizers is related to the performance of production of this genus which is limited by the unique, long, and complex life cycle of *Streptomyces* species, which produce as one of two distinct filamentous cell forms. They first form vegetative hyphae firmly attached to the growth substrate and subsequently develop aerial hyphae, which differentiate into long-chain prespore compartments that subsequently mature into individual spores (Bush et al., 2015). Khushboo et al. (2022) indicated that the growth of *Streptomyces* in liquid media by large scale production may reaches up to pellet formation starting by development of hyphae instead of spore formation, while formation of spores and aerial mycelium become blocked in various *Streptomyces* strains. Physiological conditions including pH, medium composition and the stirring speed of the reactor should be considered to regulate mycelia morphology.

Another difficulty in handling *Streptomyces* is the contamination which could occur specially during the early growth phase (Shepherd et al., 2010). Moreover, there are also lack available data on *Streptomyces*-based biofertilizer interaction with soil microbial communities, probably due to the high number of experiment under controlled conditions rather than under field (Pacios-Michelena et al., 2021).

Thus, to highlight the advantage of *Streptomyces* at the commercial level, more studies on growth parameters are needed such as formulating novel media, defining the optimal conditions of each species, designing a simplified fermentation process, ensuring their purity to achieve higher production in less time and reduce production cost. Moreover, extensive field experiments are necessary to better understand their complex relations with plants varieties, soil microbial communities and environmental conditions to ensure their persistence in soil and produce an efficient and competitive PSS-based biofertilizer.

## Conclusions and perspectives

*Streptomyces* species have been reported to be effective in various ways that naturally improve plant growth and development. The present review highlighted the potential of *Streptomyces* as a P solubilizer, as it demonstrated the mechanisms these bacteria employ to solubilize different P sources, as well as their involvement in other activities; their various plant growth-promoting traits; and their ability to act as biofertilizers, bioregulators and biocontrol agents. All these qualities appear to characterize the members of this genus and play the most important role in increasing agricultural productivity. Moreover, this review showed that the numerous studies on selection and characterization and numerous greenhouse experiments that have been performed until now are still insufficient. What is clear, though, is that PSS play an important role in the mobilization of soil P, while no studies have reported PSS to be commercial biofertilizers or demonstrated their potential effects under field conditions. For these reasons, we suggest that i) vigorous research at the field level is needed to focus on the use of *Streptomyces* in crop production, ii) that studies on their association effects under different crop species and in different environmental conditions are need, and that iii) there is also a need for further research to better understand how it could be possible to develop potential multifunctional bioformulations with PSS as biofertilizers.

## Author contributions

FC drafted the manuscript. VV reviewed and editing the manuscript, conceived the concepts of the study and participated in its design. OP reviewed the manuscript and contributed to its conceiving. All authors contributed to the article and approved the submitted version.
